# Evaluation of the ventriculocholecystic shunt—an overview of present practice in adult and pediatric hydrocephalus

**DOI:** 10.1007/s10143-021-01472-x

**Published:** 2021-01-22

**Authors:** Cezar Octavian Morosanu, Adelina Priscu, Ioan Stefan Florian

**Affiliations:** 1 Department of Neurosurgery, Southmead Hospital, Bristol, UK; 2 Department of Internal Medicine, Indiana University Health Ball Memorial Hospital, Muncie, IN USA; 3 Department of Neurosurgery, Cluj County Hospital, Cluj-Napoca, Romania

**Keywords:** Hydrocephalus, Gallbladder, Ventriculocholecystic, Biliary shunt

## Abstract

In the context of hydrocephalus, there are a multitude of therapeutic options that can be explored in order to improve patient outcomes. Although the peritoneum is the current elected clinical solution, various receptacles have been utilized when experiencing contraindications. Along with the ventriculoatrial or ventriculopleural, the ventriculocholecystic shunt was also described as an alternative. In order to make a decision on a place for drainage, the surgeon must be knowledgeable on details from modern literature. The main target of this review was to summarize the currently available information on this topic and assess the status of the gallbladder as a viable option for cerebrospinal fluid diversion.

## Introduction

The ventriculocholecystic shunt (VCS), also called a ventriculo-gallbladder or ventriculobiliary shunt, was first designed by Smith et al. [63] and represents one of the 36 options of receptacles that have been explored for the purpose of adult and pediatric hydrocephalus treatment [43]. When dealing with this neurosurgical pathology, the first treatment choice of cerebrospinal fluid (CSF) diversion is the ventriculoperitoneal (VP) shunt, but in some instances, the device is rendered unfeasible due to various complications. These clinical contraindications steer the therapeutic decision towards other anatomical sites. If the peritoneum cannot support a shunt, the atrium is considered the next viable option. This propensity of electing the ventriculoatrial (VA) shunt as a second option is not guided necessarily by a standard protocol decision, but by the surgeons’ experience in this direction. With the exception of the atrial and pleural shunts, choosing one shunt over another has never undergone a rigorous statistically significant comparison, but has been generated only through the inability of performing a previously mentioned option. The VCS has only been tested when the peritoneum, atrium, or pleura failed in delivering a suitable therapeutic response. At this point, there are no guidelines that can regard the VCS as an immediate solution after the VP shunt, despite the large amount of pertinent studies that have delineated this technique.

The target of this review is to create a full view of the situations in which the VCS has been used for the past 50 years, along with associated physiology that could make it a proper biological candidate for CSF shunting, as well as the complications that could impair its usage in the long term. The questions raised by this review are related to the usefulness of this type of shunt in modern practice aiming to explore the hypothesis that the gallbladder might be a viable cavity that can indirectly relieve intracranial pressure.

## Physiology of gallbladder shunting

There are several aspects that need to be taken into consideration in relation to the underlying physiology governing a proper functioning of a VCS. This involves the mechanisms of storing and secreting the excess fluid in the gallbladder, the size of the gallbladder, the intracholecystic pressure, the intraventricular pressure, the difference between the two, and the volume of CSF coming through the shunt.

The gallbladder provides the secretion of bile necessary in gastrointestinal processes. Bile originates in the hepatocytes and consists mainly of water (95%) and only a small proportion is represented by bile salts, cholesterol, amino acids, phospholipids, bilirubin, and steroids [10]. In the case of a cholecystic shunt, the gallbladder is also responsible for eliminating CSF, done through the relaxation of the sphincter of Oddi.

In the case of the peritoneum or the pleura [33], the principle is to allow the absorption of CSF, whereas for the gallbladder, the main process is storing fluids in order to secrete them in another compartment from where they are later eliminated. The urinary bladder uses the same mechanism in the ventriculovesical shunt [5], but the difference is that the gallbladder can also absorb certain elements such as water, electrolytes [58] and other organic substances [45], whereas the urinary bladder is impermeable [44]. Despite this constituting a minor advantage, the patency of a VCS should rely on the eliminating properties of the gallbladder and not so much on its absorptive capacity. The pH of the gallbladder content should not be the subject of major alteration given that both fluids have a pH ranging from 7 to 8, although there are various elements that can influence the biochemistry of the CSF [7] as well as of the bile [39].

The size of gallbladder is also important as it is necessary to know how much fluid this type of cavity can store. The size increases with age and can be dependent on the type of oral intake [65]. In pediatric patients, gallbladder size can vary from 1.4 cm^3^ in patients less than 1 year old, to 11.1 c.m^3^ in patients up to 16 years old [72]. It is difficult to state how much extra CSF fluid can the gallbladder accommodate, but smaller gallbladders could constitute an issue. Despite this aspect, the majority of VCSs in literature have been performed on children (Table 1).

**Table 1:** Pediatric hydrocephalus

Year	Author	Number of patients	Age	Previous shunt	Follow-up	Complications
1959	Smith et al.	10	Not specified	Not specified	Not specified	4—died 6—no complications
1985	Berstein et al.	1	5 y	VA, VP	n/a	Death due to neurogenic pulmonary edema with biliary ventriculitis after < 24 h of shunt placement
1987	West et al.	25	6 mo–16 y	VP, VA 1—none	4 mo–9 y	Proximal/distal shunt obstruction/infection, biliary tract infection, subdural hematoma, gallbladder atony, ventriculo–small bowel fistula
1993	Stringel et al.	8	8 w–15 y	VP, VA	10 mo–8 y	5—no complications 3—complications (distal end malfunction, shunt infection, gallbladder atony)
1997	Ketoff et al.	16 (not specified how many were children)	0.9–23 y	VP, VA, VPL, VSaph, 1—none	4 w–7.5 y	9—no complications 7—complications (shunt obstruction/disconnection/infection, enterotomy, pseudomembranous colitis, subdural hematoma, slit ventricles, cellulitis, wound dehiscence, unilateral hydrocephalus from undrained ventricle contralateral to ventriculostomy)
1997	Novelli et al.	6	Not specified	VP, VA, VPL	8 mo–8 y	No complications
2000	Rajaraman et al.	1	5 y	VP	n/a	Distal end obstruction (retained connector in the gallbladder)
2000	Frim et al.	1	4 y	VP	Not specified	Not specified
2005	Hamamcioglu et al. [23]	1	10 y	VP, VPL	Not specified	Not specified
2005	Olavarria et al.	4	~ 8–12 mo	VP, VA	3.5–4y	1—no complications 3—complications (bile reflux, wound infection following laparotomy for abdominal pain, unspecified distal shunt malfunction)
2006	Surfield et al.	1	7 y	VP	11 y	Cholelithiasis encrusted on shunt tubing at 18y, VCS revision, then symptom-free at 3 mo follow-up
2007	Pal et al.	2	3½ y, 6 mo	VP, 1—none	2 ½ y, 3 y	No complications
2007	Weinzierl et al.	2	~ 6 mo, ~ 9 mo	VP, VA	2 y, 3 y	Death due to distal shunt occlusion, Post-prandial headaches after ingestion of fatty foods, but well-functioning VCS
2008	Aldana et al.	18	4 mo–17 y	17–VP, VJ, 1—none	1 w–8.5 y	11—no complications 7—complications (shunt infection, proximal/distal malfunctions, “sludge” in the biliary duct and the CBD, gallbladder stones, cholecystitis)
2009	Girotti et al.	2	3 y, 12 y	VP, VA	2 y, 9 y	No complications
2010	Polo et al.	4	4–13 y	VP, VA	10 mo–3 y	3—no complications 1—Acalculous cholecystitis treated medically, then prolonged fever initially thought to be ventriculitis, but later proved Silastic allergy—VCS was removed
2011	Weiner et al.	1	13 mo	VP	n/a	MRSA Ventriculitis
2012	Lyngdoh et al.	2	8 y, 11 y	VP, ETV	3.4 y	No complications (however hydrops noticed on follow-up MR cholangiogram)
2012	Sepulveda et al. [59]	1	Not specified	VP	18 mo	No complications
2013	Demetriades et al.	2	9 mo, 3 ½ y	ETV, VP	46 mo, 28 mo	No complications
2013	Parikh et al.	1	Not specified	VP, VA	n/a	2 episodes of ascending cholangitis, renal failure, and sepsis at 26 y, secondary to a retained metallic fragment of a VCS in the common bile duct
2013	Shakir et al.	1	9 mo	Ventriculosubgaleal	Not specified	Removal of the shunt after an infection contracted during a proximal revision procedure
2013	Woodfield et al.	1	1 y	VP	2 y	CSF overproduction which exceeded the gallbladder capacity
2014	Kulwin et al.	1	9 y	VP	Not specified	Bile peritonitis due to shunt fracture
2015	Rivero-Garvia et al.	3	7 y, 16 mo, 4 y	VP, ETV, VA, Vfem	45 mo, 14 mo, 27 mo	Case 1—No complication Case 2—Valve infection that required conversion to VP shunt. Died 14mo later of atypical pneumonia Case 3—Disconnected biliary catheter 6mo after placement
2019	Henderson et al.	3	2 y, 2 y, 8 y	VP, VA, ETV	22 mo, 12 mo, 1—none	2—no complications 1—shunt dysfunction (VCS failed due to high volume of CSF, overwhelming the drainage capacity of the gallbladder)
2019	Pancucci et al.	1	4 mo	None	14 mo	No complications
2019	Ignacio et al.	1	20 mo	VP	3 y	No complications
2020	Alraee et al.	1	11 y	VP	1 y	Gallbladder stones

The first experiments with VCS were performed by Smith et al. (1959) on a dog model [63]. Smith’s initial feasibility study determined that the intracholecystic pressure of 10–20 cmH_2_O would maintain a satisfactory intracranial pressure and that the lytic action of the bile would prevent any fibrinous adhesions at the distal segment of the shunt. The physiology of the canine biliary tract does not present significant differences from that of a human [53].

In addition to the already added inner gallbladder pressure, intra-abdominal pressure (IAP) might also play an unfavorable role and increase the intracholecystic pressure. In the case of ventriculoperitoneal shunt, IAP has been shown to increase the risk of shunt failure by contributing to an imbalance in the flow of the CSF [42]. Although no proper studies have been performed to objectively measure this aspect, it is reasonable to suspect that the intracholecystic pressure might be subject to the same physiological process as the ventriculoperitoneal shunt.

Intraventricular pressure (IVP) dynamics in VCS has been studied by Frim et al. in relation to food ingestion and their observations revealed that in the post-prandial period the IVP of the 4-year-old patient rose by 13 cmH_2_O with a maximum after 75 min. In patients that require relatively elevated intraventricular pressures to prevent over-drainage phenomena, which can present with chronic headaches or a subdural hematoma, this can be a good thing. Despite having only one patient in their telemetric study, and not a cohort of patients, the authors draw attention to an important issue, that feeding can have an impact on the VCS pressure dynamics [18].

Another aspect highlighted by Henderson et al. is that CSF output in an EVD can have the potential to indicate the prognosis of a shunt. In their experience, the patient who had higher volumes of CSF recorded in the pre-shunt EVD resulted in shunt failure, whereas the cases with lower EVD volumes had a significantly better outcome [26]. Furthermore, Woodfield et al. recommend considering a VA shunt when large volumes of CSF require drainage after managing a pediatric case with craniopharyngioma and CSF overproduction. The patient initially had a VP shunt, which proved to be unable to absorb the increased quantity of fluid, causing the child to develop CSF ascites later on. Then, the next therapeutic option was a VCS, which also failed, suggesting, as Woodfield et al. state, that “in cases where peritoneal absorption has failed due to large volumes of CSF, the gallbladder may not be a suitable alternative” [71].

It should be mentioned that the gallbladder was used on very rare occasions as the site of CSF diversion coming from the lumbar spine in the so-called lumbar-gallbladder shunts. Complications such as chemical meningitis from bile reflux are reported in literature, which is a potential complication of cranial diversions, as well [8]. This raises the question of the biochemical effect that bile has on the cerebral tissue. Although bile is a relatively sterile substance [13, 29], leakage into the peritoneum generates an inflammatory reaction in the form of serous peritonitis [1]. Experimental studies have demonstrated that this is a result of the effect of bile salts rather than bilirubin; in addition, bile also stimulates lipopolysaccharide release enhancing the peritoneal inflammatory response [6]. It is highly possible that the meninges could be subject to the same process. In the case presented by Barami et al., the high acidity of the bile was also incriminated for the patient developing aseptic meningitis, encephalopathy, and severe lumbar arachnoiditis [8]. In the case reported by Bernstein, the effect of bile on the brain was evaluated during the autopsy. This determined the histological changes of brain saponification and emulsification secondary to this type of injury—which appear to be quite different from the normal appearance of kernicterus. Their findings show that bile caused acute cerebral edema with diffuse neuronal anoxic changes in a pseudolaminar pattern. The cerebellum had degenerative changes in the Purkinje cells and dentate nuclei, the thalamus, and hypothalamus displayed astrocytosis and gliosis and the cerebral arachnoid had macrophages with bile pigment [9].

## Technique and perioperative aspects

The first technique of VCS was proposed by Smith et al. who performed it on a series of 10 patients, recording a mortality rate of 40%. According to them, the patients did not die of causes related to the actual shunt, but to other comorbidities [63]. The VCS technique has also been attributed to Luis Yarzagaray (1958), but despite his 15 cases being mentioned in the works of Anthony Raimondi, he did not have any personally published observations [54]. Since then, advancements in the operative room and antibiotic therapy have improved patient outcomes. In the preoperative assessment, biliary studies (liver function tests and cholangiography) should be considered, as well as abdominal ultrasound, to identify if any pre-existing biliary pathology would contraindicate a VCS. James et al. emphasize the role of preoperative gallbladder imaging, such as abdominal CT or ultrasonography. Their database includes a case that was considered for VCS, but had gallbladder agenesis and a case that had initial sludge formation identified on ultrasound, which eventually led to shunt blockage and removal [32]. As indicated by Henderson et al., careful consideration should also be made in patients with high EVD drainage levels [26].

Due to the hepatobiliary involvement, the help of a general surgeon, or a pediatric surgeon if the patient is a child, would be a useful addition to the neurosurgical operating room staff. After Smith et al., Bernstein et al. reported the case of a 5-year-old who underwent VCS insertion after multiple other shunting attempts. The proximal component of the shunt was always placed in the ventricles using the standard technique. For the distal component, the procedure commenced via a right subcostal incision, which allows access to the fundus of the gallbladder and visualization of the distal segment of the shunt. The distal end of a Raimondi one-piece catheter was used and a cholecystostomy was made in the dome of the gallbladder. A catheter with a low-pressure valve was inserted and afterwards checked for any signs of leakage. Unfortunately, the patient did not have a favorable outcome, but this was the first attempt made by anyone at this procedure after Smith/Yarzagaray [9].

**Fig. 1:**
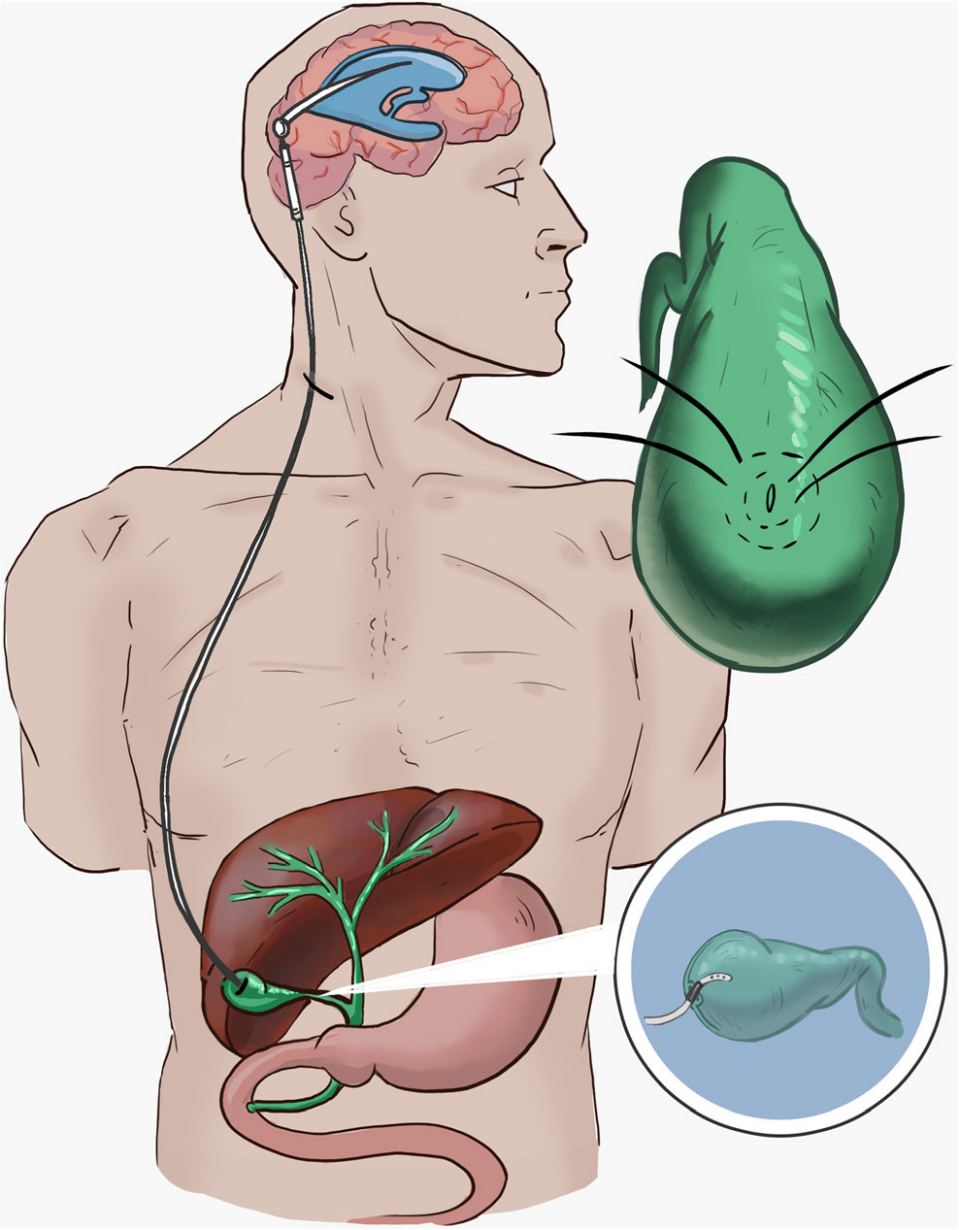
Ventriculocholecystic shunt. Metal connector inserted in gallbladder and secured with a concentric purse string suture

The technique, later coined the *Yarzagaray technique* by Raimondi [54], remained relatively the same over the years with very slight alterations reported in literature, in terms of technique and post-operative care (Fig. 1.). After isolating the gallbladder, West et al. put 2 concentric purse-string 3–0 nonabsorbable sutures, and a small incision was made in it. Bile was extracted in a minor quantity on this occasion. The distal end of the shunt, equipped with a low-pressure one-way valve, was coiled in a non-obstructive pattern over the dome of the right hepatic lobe and then inserted in the gallbladder where the purse strings were used to secure the catheter at the level of the metal connector [70]. After ensuring that the distal catheter is properly positioned in the cavity of the gallbladder, Girotti et al. used the ends of the purse-string sutures to attach them to the anterior peritoneum [21]. Lyngdoh et al. brought an alteration to the Yarzagaray method by inserting up to 7 cm of the distal catheter in the cavity of the gallbladder. He did so by attaching the tube around the connecter to the gallbladder serosa and using 2 purse strings [38]. Aldana et al. sectioned the distal catheter 2.5–3 cm from the end and attached it to a Holter type A nonferromagnetic straight metal connector which eventually was introduced in the gallbladder [2]. Ketoff et al. used a multiperforated tapered silastic catheter (Dow Corning, Midland, MI) inserted in the gallbladder [34], and Olavarria et al. and Shakir et al. specify the barium impregnated nature of the distal tubing [47, 60].

The length of the segment of the distal catheter which is inserted in the gallbladder also seems to vary. In most cases, around 4–5 cm of the tube was placed in the cavity; however, West et al. report inserting 2 cm [70], Girotti et al. 6 cm [21], and Lyngdoh et al. 7 cm [38]. Despite these variations, there has been no particular documented correlation between this aspect and shunt prognosis.

In addition, Aldana et al. also described their type of antimicrobial prophylaxis by using one preoperative intravenous dose of cefazolin (25 mg/kg) and an intraoperative bacitracin solution (50,000 U in 500 ml of saline) for cavity irrigation [2]. In the case of Rajaraman et al., to test for any signs of leakage of the VCS, a radionuclide cholescintigraphy scan was performed post-operatively [55], whereas Lyngdoh et al. used an MRI cholangiogram to identify any potential postsurgical issues [38].

More recent descriptions of the technique have been done by Matushita [41] and by Fulkerson et al. in Cohen-Gadol’s Neurosurgical Atlas, in which the technique follows most of the steps of the original procedure [19]. However, over the many decades since the first intervention was performed, various valve systems and components of shunt systems have been introduced in clinical routine, making it difficult to draw specific conclusions on VCS efficiency.

Unidirectional valves have been used in all cases of VCSs with the intention of preventing bile reflux. Despite this property, a few cases have been reported where bile managed to ascend, causing bilious ventriculitis in one case and death due to saponification and emulsification in another case. The valve was tested before the intervention and after the complication and the reflux system seemed to be patent. It has been hypothesized that increased abdominal pressure caused by coughing after the surgery could have generated a disruption in the valve mechanism and this could have led it to fail. Furthermore, the majority of authors advocate for a low-pressure valve as the increased intracholecystic pressure during contraction contributes to the valve pressure. West et al. reports using *very* low-pressure one-way valves in most cases justifying that the intrinsic biliary resistance maintained appropriate intracranial pressure [70]. Ketoff et al. and Girotti et al. on the other hand use a medium pressure valve with good results [21, 34]. Olavarria et al. specify using a Delta 1 valve [47], Aldana mention both Delta and Holter valves in their protocol [2], whereas Lyngdoh et al. used a Chabbra shunt which is designed with two slit valves [38].

To minimize the chance of complications, a one-way, low-pressure valve is thus proposed by most cases in literature and these seem to be fit for purpose, but further research could reveal the potential of other types of valves.

Apart from the classical open subcostal approach to insert the distal end of the shunt, several other techniques have been documented. Percutaneous insertion of a VCS was also reported in a 9-month-old with hydrocephalus due to a grade 4 intraventricular hemorrhage [60]. Furthermore, Pancucci et al. were the first to describe the successful positioning of a VCS with the assistance of laparoscopic surgery in a 4-month-old patient with hydrocephalus secondary to an optic chiasmal hypothalamic glioma [49]. Ignacio et al. also used this approach in a 20-month-old patient with increased CSF levels due to a grade 3 ventricular hemorrhage [28]. In the case presented by Pancucci et al., the technique was done with the help of a pediatric surgeon through a three-port laparoscopic approach, and a 7-French pigtail catheter was inserted in the fundus of the gallbladder via the Seldinger technique. Their surgical intervention was based on a study by Soleman et al. on laparoscopic insertion of ventriculoperitoneal shunts [64]. The technique appears to have been better tolerated than the subcostal approach, and the patient was complication-free at 14 months of follow-up.

A particularity of the distal catheter in children is allowing a sufficient length of tubing in the abdomen to adapt to the growth of the patient. The issue of the length of the VCS distal catheter has not been thoroughly discussed in literature. Stringel et al. and Olavarria et al. placed around 30–40 cm of redundant catheter in the abdomen to facilitate this aspect [47, 66], whereas Novelli et al. inserted around 15–20 cm [46]. Some authors mention adding excess redundant catheter [2, 48, 70], but do not specify the exact length. Extended tubing is generally well tolerated in children with VP shunts [12] and this progressively uncoils as the child grows. This would occur in a similar way in a pediatric patient with a VCS inserted either classically or laparoscopically. A few potential consequences can occur if the distal catheter does not have an adequate length. As the patient grows, there would be increasing tension applied to the site where the shunt is inserted in the gallbladder. This can lead to the dislodgement of the shunt from the cholecystic compartment. Subsequently, not only would the CSF be diverted into the peritoneal cavity but bile would also leak into the abdomen. This can cause serious complications such as bile peritonitis as described by Kulwin et al. [35]. Some authors have also reported a pediatric case where the straight connector was retained in the gallbladder after the shunt was disconnected [55]. Fortunately, their patient did not show any form of leakage, but in these cases, it would be important to inspect the cholecystic compartment as well and close it if necessary, in order to avoid the risk of bile leak in the abdomen.

## VCS in Adult Hydrocephalus

There have been 6 documented adult patients, between the ages of 20 to 46 years, that have undergone VCS for hydrocephalus of different etiologies (Table 1). The number of adult patients is potentially higher, but there is no indication as to how many patients over the age of 18 years old are in Ketoff’s patient population [34]. All cases had a previous form of the shunt that either failed multiple times or required converting to a different type of shunt. Girotti et al. illustrate the case of a 46-year-old with hydrocephalus due to several cerebral masses who underwent ventriculoperitoneal shunt insertion and required up to 4 revisions. After the failure of the ventriculoperitoneal shunt, a VPL shunt was inserted but failed as well due to inappropriate absorption. The final decision was to resort to a VCS, which was found to be very effective at 1-year follow-up [21]. Guclu et al. go even further and perceive VCS as a salvage procedure in their case report, where other VP shunt and VA shunts have been unsuccessful [22]. Despite this aspect, not all VCSs prove to be so effective.

The period in which VCSs are patent also varies. Fountas et al. describe a case of a 51-year-old who was admitted to the emergency room 20 years after having a VCS inserted. The patient was investigated, and it appears that a calculus formed around the distal end of the shunt and had to be removed along with the entire gallbladder in a cholecystectomy intervention. Subsequently, the shunt was converted to a VPL shunt. Despite the unfortunate outcome, this case describes the longest period of time in which a VCS was patent [17].

It appears that very few adult cases can be found in literature, and databases can be inconsistent, lacking certain details, such as follow-up times and complication rates. Therefore, statistical analysis is difficult to construct.

## VCS in Pediatric Hydrocephalus

The majority of cases discussed in literature about VCS involve pediatric patients. A total of 105 patients have been identified in 29 articles comprising 4 larger case series (Table 2). On top of that, the case series of 15 patients attributed to Luis Yarzagaray could potentially be included, adding up to a total of 120 patients. Given that the work of Yarzagaray, L. has never been published and only mentioned in Raimondi’s Pediatric Neurosurgery Textbook, it is difficult to incorporate them without any proper details. Furthermore, some authors fail to report the age of the patients, thus it is difficult to include them in any of the adult or pediatric populations [36, 54].

**Table 2:** Adult hydrocephalus

Year	Author	Number of patients	Age	Previous shunt	Follow-up	Complications
1997	Ketoff et al.	16 (not specified how many were adults)	0.9–23 y	VP, VA, VPL, VSaph 1—none	4w–7.5 y	9—no complications 7—complications (shunt obstruction/disconnection/infection, enterotomy, pseudomembranous colitis, subdural hematoma, slit ventricles, cellulitis, wound dehiscence, unilateral hydrocephalus from an undrained ventricle contralateral to ventriculostomy)
2007	Fountas et al.	1	31 y	Not specified	20 y	Cholelithiasis
2009	Girotti et al.	1	46 y	VP, VPL	1 y	No complications
2010	Polo et al. [52]	1	33 y	VP	10 mo–3 y?	No complications
2016	Hasslacher-Arellano et al. [25]	1	27 y	VP	Not specified	Not specified
2017	Guclu et al.	1	44 y	VP, VA	Not specified	Not specified
2017	Scaife et al.	1	20 y	VP, VPL, VA	Not specified	Ascending cholangitis due to a retained fragment in the gallbladder which later migrated to CBD and got lodged in the ampulla

Other CSF derivations have been sought before performing a VCS. Most authors report multiple attempts on VP or VA shunts. On a few occasions, more infrequent shunts have been mentioned as previous options, such as ventriculosaphenous [34], ventriculofemoral [56], or ventriculojugular shunt [2]. West described patients that had up to 6 revisions of their VP/VA shunts [70]. However, 5 studies presented cases that had a VCS as a first line of treatment. Pal et al. report the case of a 6-month-old patient with postmeningitis hydrocephalus shunted with VCS [48]. The initial plan was to perform a VP shunt, but after uncovering the extent of the intra-abdominal adhesions, the therapeutic decision was shifted to a VCS. Ketoff et al. and Aldana et al. each have a patient for which VCS was the first intent, one of the reasons being previous abdominal surgery [2, 34].

The largest case series up to this date remains West et al. with 25 patients over a period of 16 years. The patients’ ages varied between 6 months and 16 years and included congenital hydrocephalus or hydrocephalus secondary to myelomeningocele, meningitis, cerebral masses, and intraventricular hemorrhage. All except one had previous failed VP and VA shunts. In this series, three patients deceased in the following 4 to 24 months due to unrelated causes. Despite some manageable complications, the authors report good outcomes in the majority of the remaining patients; 14 having the VCS in place long-term, among which 6 even for up to 9 years [70]. The second largest case series is a more recent study belonging to Aldana et al. with a total of 18 patients, 61% of which did not have any immediate shunt complications. In a long-term follow-up lasting between 1 and 8.5 years, 72% of cases had functional shunts (13 out of 18) [2]. Ketoff et al. also had good results with a larger case series of 16 patients, out of which 11 were still working at a median follow-up rate of 3 years, thus a complication rate of 43.7% [34]. Stringel et al. reported a slightly lower complication rate of 37.5% [66].

There have been a number of case reports and case series (8 out of 29) that have presented no complications. Novelli et al. did not identify any issues with the VCS in any of their 6 patients. They had a mean follow-up period of 32 months, some shunts being patent up to 8 years [46].

The large numbers of VCS reported in literature are case reports with various particularities, from etiology and technique to complications and outcomes. In the case of optic chiasmal hypothalamic astrocytoma (OCHA), there have been some reports in which VCS was a suitable option. It is a documented fact that ascites is a significant complication in OCHA patients [61], thus making the VP shunt an unfeasible option [20]. In an attempt to find an alternative, Olavarria et al. described 4 cases of OCHA treated with VCS. There were no mortalities, and all VCSs were patent for a significant amount of time. Three out of four, however, had to be converted to different types of shunts. One remained in place and had a longstanding functional VCS at 4 years of follow-up [47]. In addition, Pancucci et al. performed a laparoscopic VCS as the first line of treatment in a patient with OCHA and his results were promising, as no complication was demonstrated at 14 months of follow-up [49]. Alraee et al. also described a laparoscopic approach to place a VCS in a patient with an extensive optic glioma, which eventually developed gallbladder stones [4].

Lyngdoh et al. has treated post-tuberculous hydrocephalus with tubercular adhesive peritonitis with a VCS in 2 separate cases. Due to the difficulty of managing this pathology [62], the authors advocate for considering a VCS for treatment [38].

Another pathology in which VCS has been proposed as a solution is hydrocephalus in the context of congenital plasminogen deficiency. Weinzierl et al. reported the case of 2 pediatric patients with this hematological disorder that were initially treated unsuccessfully with a VP shunt. Given the thrombogenic nature of this disorder, VA shunts can have unfavorable consequences. The first patient was treated both with VP shunt and VA shunt and resulted in failure. Subsequently, VCS was implanted, and functioned well for 2 years, until it got occluded and eventually led to the patient’s death. The second patient was operated sooner with a VCS and has had a significantly better outcome at 3 years of follow-up. The authors suggest that early management can make a difference in this type of pathology [69]. Furthermore, Demetriades et al. describe their experience with VCS in a 9-month-old female patient with congenital plasminogen deficiency. After having a failed VP shunt, the authors did not consider VA shunt and opted for a VCS. This proved to be a practical decision and the patient had a good post-operative outcome with a functional shunt at 46 months of follow-up. Far from suggesting this procedure should constitute a standard in these clinical entities, different authors suggest taking into consideration this site as a viable alternative [14].

## Contraindications

Any biliary pathology could potentially impact the outcome of a VCS. Thus, a thorough preoperative assessment is required. It is first important to establish the presence of a gallbladder, either by identifying it via ultrasonography or by enquiring of any cholecystectomy in the past medical history.

Once visualized, imagistic investigation would help in recognizing any issues. An intrahepatic or hypoplastic gallbladder would be a contraindication as it would not be able to accommodate larger volumes of fluid.

Biliary infections such as cholecystitis, gallbladder abscesses, or predisposing factors to cholelithiasis, such as hemolytic anemias (sickle cell disease, hereditary spherocytosis) would impair VCS function.

According to Alraee et al., the presence of sludge in the gallbladder, without any signs of inflammation or biliary duct dilation, does not represent a contraindication. They managed a pediatric case of hydrocephalus secondary to optic glioma, in which a VCS was inserted in a child with known biliary sludge. The sludge was removed prior to shunt placement by irrigating the gallbladder with normal saline. Eventually, the patient developed jaundice and was diagnosed with cholecystitis and cholangitis. The case was managed by performing an ERCP with sphincterotomy and stone extraction without cholecystectomy or shunt removal, and the child was complication-free at 12 months of follow-up [4]. This is in contrast with James et al. who resorted to shunt removal [32].

Ignacio et al. delineate a list of contraindications in their decision algorithm for VCS placement. As their study is focused on laparoscopic placement, the inability of tolerating a pneumoperitoneum is also mentioned. General contraindications such as severe abdominal adhesions would also be a criterion, such as in the case of a VP shunt [28].

## Specific VCS complications

In terms of complications, various authors have documented aspects that are common to all shunts, such as shunt blockage or infection, but also details that were specific to VCS. Bernstein et al. outline a fatality case of biliary ventriculitis secondary to a gallbladder shunt. The report written in 1985 appears to be the only case that succumbed as a direct result of the shunt system [9]. Kulwin et al. report the case of a patient with a gallbladder shunt that developed bile peritonitis due to a fracture of the catheter below the valve and required to be removed and converted to a VA shunt [35]. MRSA ventriculitis has been cited in a 13-month-old patient who presented septic to the hospital. She was initially started on intravenous vancomycin but did not show signs of improvement, so intraventricular administration was commenced. Improvement was noted, but the shunt had to be converted to a ventriculoureteral one [68]. To support this approach, most experts recommend combined intraventricular (IVT) and IV antibiotic therapy. They also recommend removing the shunt and placing an EVD, rather than performing externalization of the infected shunt [11, 30, 31].

Although not reported, Demetriades et al. also remark that cholecystitis can potentially yield ascending and descending VCS infections [56] as it has been observed in VP shunts [40].

Specific mechanical complications include obstruction due to retained distal metal connector after a VCS was removed as shown by Parikh et al. The 26-year-old patient suffered 2 acute cholangitis attacks and renal failure as a result of sepsis so she eventually had to undergo cholecystectomy and the removal of the obstruction from the common bile duct [50]. Another case of cholangitis due to a retained catheter fragment was reported by Scaife et al. in a patient that developed symptoms several years after VCS removal [57]. Obstruction can also be caused by a calculus formation around the distal end of an existing shunt. Fountas describes cholelithiasis in a longstanding shunt that eventually led to it being removed through a cholecystectomy [17], whereas Alraee et al. performed the stone extraction through an endoscopic retrograde cholangiopancreatography (ERCP) with sphincterotomy, without shunt removal [4]. Surfield and Klein also identify the same issue in an adult patient [67].

Rajaraman et al. draw attention to a general state of awareness when dealing with a VCS. As it is not so commonly used, radiologists can confuse the VCS to a complication of a VP shunt in the absence of an accurate clinical history [55].

Gallbladder atony has also been documented by West et al. in 3 children 3–13 months following shunt placement, but the issue resolved after intravenous administration of cholecystokinin. Narcotic medication and Oddian spasm were suspected to be the underlying mechanism of this disturbance [70].

Intra-abdominal complications such as gastric [3] or intestinal perforation [73] have been frequently reported in VP shunts. In cases of biliary CSF diversion, Ketoff et al. reported a patient who had an enterotomy and pseudomembranous colitis post-VCS insertion [34], and West et al. had one patient in their case series who experienced a small bowel fistula due to a VCS [70]. In addition, since the bile is diluted with CSF, there is a possibility this could impact its capacity to emulsify lipids. Although not an aspect that has been properly investigated, it is worth taking this possibility into account and making adequate adjustments to the patient diet if necessary.

Another complication that has been evaluated by Frim et al. is the frequent post-prandial headaches that can occur in some patients. This aspect was quantified in a noninvasive telemetric study and found that the reason for this is increased intraventricular pressure after having a meal. The study was performed on only one patient, so it is difficult to assess whether it is caused by all types of feeding or only by fatty meals [18].

## Future perspectives

There does not seem to be a trend in terms of what type of hydrocephalus can be shunted with a VCS. These types of shunts have been evaluated in various instances when other shunts failed and have rendered good results. Various studies have compared VP and VA shunts and have concluded that there is variably no difference between the complication rate of the two [27], but no comparison has been made between case series of VP or VA shunts and VCS. VP shunts have been subjected to statistically relevant comparisons to other types of shunts such as the VPL shunt [51] or VA shunt [37] and concluded that there are equally as effective as the first option. When trying to choose a second option after a VP shunt has failed, careful consideration should be made to the complications that can occur in patients. In this matter, there is a significant difference in what complications can occur from VA [24] shunts when compared to VCSs. Arguably, VCSs are significantly less at risk of causing a life-threatening issue, compared to the risks that can occur from the migration, thrombosis, malposition, or infection of a shunt in the atrium. Despite this aspect, it is important to note that relevant comparisons are difficult to make, as the number of patients who underwent VC shunting is considerably smaller.

There is no established timeline of follow-up for VCS patients, some of the cases being reviewed early due to potential complications. Given that this technique is not widely performed and complications have been reported even after 1 week, it would be advisable to have early follow-up with adequate imaging and blood tests.

For follow-up purposes, shunt X-ray series are a very useful investigation for VP shunts [15] and these could prove relevant for VCSs as well, particularly the abdominal X-ray to assess the distal component. Ultrasonography is an invaluable resource for biliary evaluation [16] and this could be performed both immediately post-op to ensure proper gallbladder emptying as Aldana et al. suggest as well as routinely in an outpatient setting [2]. In situations where the ultrasound assessment is not satisfactory, CT imaging can be used for further evaluation. Some authors such as Lyngdoh used MR cholangiography at follow-up [38]. Blood tests should also be performed and aim at evaluating for any sign of infection and or abnormality in liver function tests.

VCSs have shown promising results in patients with congenital plasminogen deficiency, post-tuberculous hydrocephalus, and OCHA patients, but further research must be conducted on larger populations before they are acknowledged as a viable alternative. It would be difficult to recommend VCS as first-line treatment in these cases, but it should definitely be taken into account given that there are studies in literature that suggest its effectiveness.

There is a significant interstudy variability among current literature regarding VCS outcomes. Girotti et al. conducted a review in 2009 on the largest VCS case series at that point, and found a percentage of 63% of patients that benefited from a long-term good outcome. These results would be comparable to VP or VA shunts, but further analysis is required to clarify different aspects regarding indication. It is also difficult to statistically evaluate as there is quite an inconsistency in data in terms of the number of previous shunting procedures or follow-up time. Also, there are a significantly lower number of cases compared to other diversion options [21].

## Conclusion

In neurosurgical literature, ventriculocholecystic shunts seem to have shown encouraging results in both adults and children. There are several case series that exhibit results comparable to ventriculoperitoneal or ventriculoatrial shunts and there are indications that they could be a feasible option in congenital plasminogen deficiency, optic chiasmal hypothalamic astrocytomas, and post-tuberculous hydrocephalus. Some authors advocate for VCS as an early alternative; however, there are many questions regarding this CSF diversion that are open and difficult to answer, mainly because of the small number of patients treated with this method up until now. VCS cannot be viewed as a first-line strategy as there are still aspects regarding indications, contraindications, and follow-up that require clarification. It is quintessential that a surgeon is faced with all the current facts before deciding to undertake such a procedure that can prove as challenging as other shunting methods. Further research with larger patient populations and with rigorous statistical parameters are necessary to establish the current role of the ventriculocholecystic shunt.

## Data Availability

Not applicable.
